# Prevalence of familial hypercholesterolemia in patients with premature myocardial infarction

**DOI:** 10.1002/clc.23154

**Published:** 2019-02-19

**Authors:** Yuxia Cui, Sufang Li, Feng Zhang, Junxian Song, Chongyou Lee, Manyan Wu, Hong Chen

**Affiliations:** ^1^ Department of Cardiology Peking University People's Hospital Beijing China; ^2^ Beijing Key Laboratory of Early Prediction and Intervention of Acute Myocardial Infarction Peking University People's Hospital Beijing China; ^3^ Center for Cardiovascular Translational Research Peking University People's Hospital Beijing China

**Keywords:** familial hypercholesterolemia, gene mutation, premature myocardial infarction, treatment

## Abstract

**Background:**

Familial hypercholesterolemia (FH) is a genetic cause of premature myocardial infarction (PMI). Early diagnosis of FH is critical for prognosis.

**Hypothesis:**

To investigate the prevalence of FH among a cohort of Chinese patients with PMI using genetic testing, and to evaluate different diagnostic criteria.

**Methods:**

A total of 225 consecutive PMI patients were recruited. Low‐density lipoprotein receptor (LDLR), apolipoprotein B (APOB), proprotein convertase subtilisin‐kexin type 9 (PCSK9) and low‐density lipoprotein receptor adaptor protein 1 (LDLRAP1) genes were detected by Sanger sequencing. FH was diagnosed using the Dutch Lipid Clinic Network (DLCN) criteria and modified DLCN criteria, respectively. The prevalence and clinical features of FH were analyzed.

**Results:**

In all PMI patients, pathogenic mutations of LDLR, APOB, PCSK9 and LDLRAP1 genes were found in 10 of 225 patients. Among all mutations, four mutations (LDLR c.129G>C, LDLR c.1867A>T, LDLRAP1 c.65G>C, and LDLRAP1 c.274G>A) were newly discovered. The prevalence of FH diagnosed by genetic testing was 4.4%. The prevalence of definite/probable FH diagnosed by DLCN and modified DLCN criteria reached 8.0% and 23.6%, respectively, and the mutation rates were 33.3% and 12.2%, respectively. The low‐density lipo‐protein cholesterol (LDL‐C) levels in PMI patients with FH were far from goal attainment. Only one of the FH patients had LDL‐C <2.5 mmol/L, and none of them had LDL‐C <1.8 mmol/L.

**Conclusions:**

The prevalence of FH among Chinese patients with PMI appeared relatively common. Underdiagnosis and undertreatment of FH are still a big problem, which should arouse a widespread concern.

## 1 INTRODUCTION

Familial hypercholesterolemia (FH) is a genetic cause of premature myocardial infarction (PMI) due to lifelong elevated serum low‐density lipoprotein cholesterol (LDL‐C) levels.^1^ As an autosomal genetic disease, low‐density lipoprotein receptor (LDLR), apolipoprotein B (APOB), proprotein convertase subtilisin‐kexin type 9 (PCSK9) and low‐density lipoprotein receptor adaptor protein 1 (LDLRAP1) genes are the most important pathogenic genes of FH.^2^, ^3^ Mutations of LDLR, PCSK9 and APOB genes are autosomal dominant inheritance. However, LDLRAP1 gene mutations produce a very rare recessive disease known as autosomal recessive hypercholesterolemia (ARH) with a similar phenotype.^4^

It is generally believed that the prevalence of heterozygous FH is 1/500 and that of homozygous FH is 1/1000000 among the population.^5^ In special subgroups of the population, such as PMI, the prevalence of FH is often higher. It has been shown that early diagnosis of FH is critical for prognosis, and the efficiency in the diagnosis of FH has been improved as the recent technical progress in genetic testing.^6^ However, in clinical practice, early diagnosis is still difficult for many cases with FH. Due to the difference in the prevalence of FH among countries and ethnicities, and complexities in the genetic variants,^7^ there are some limitations in the current diagnostic criteria and no criteria are universally applied.^8^

In China, there are few studies analyzing FH patients using genetic analysis, and novel genetic variants identified remain scarce.^9^ The rate of timely diagnosis and treatment of FH is far from satisfaction. As PMI represents a critical clinical manifestation of patients with FH, the aim of this study was to investigate the prevalence of FH using genetic testing in a cohort of Chinese patients with PMI, and to evaluate different diagnostic criteria.

## 2 METHODS

### 2.1 Subjects

All patients with myocardial infarction (MI) who visited Peking University People's Hospital between May 1, 2015 and March 31, 2017 were enrolled. MI was defined according to the Third Universal Definition of Myocardial Infarction.^10^ Patients with PMI were included (age of the first MI onset: male ≤55 years old, and female ≤60 years old). The exclusion criteria: (a) the age at the first onset of MI: males aged >55 years old, and females aged >60 years old and (b) patients with incomplete clinical data or no blood samples The protocol was approved by the Ethics Review Board of Peking University People's Hospital (No. 2014PHB125‐01), and the written informed consent was signed by all patients.

### 2.2 Collection of clinical and laboratory data

The clinical data, including age, sex, body mass index, and family history of premature coronary heart disease (pCHD), were collected. Results of laboratory examinations such as routine blood test and biochemical test during the first 24 hours after admission were also obtained. The severity of CHD was assessed according to Gensini score system as described in the previous study.^11^ Routine blood test was performed using the XN9000 automatic blood cell analyzer (Sysmex, Kobe, Japan). Biochemical testing was performed using the 5832 biochemical analyzer (Beckman, Atlanta, GA). Family history of pCHD was defined as pCHD in males aged <55 years old or females aged <60 years old in the first‐degree relatives.

### 2.3 Diagnostic criteria for FH

FH was diagnosed using the Dutch Lipid Clinic Network (DLCN) criteria which covered personal and family history of premature atherosclerosis, physical examination and LDL‐C levels.^12^ China's modified DLCN criteria proposed for FH diagnosis of the Chinese population were also employed.^13^ Untreated LDL‐C levels of individuals who were on lipid‐lowering medications but had no pretreatment LDL‐C data were conservatively adjusted by relative correction factors dependent on the dose and potency of statins. The correction factors were originated from the analysis of 71 original articles that were collated before establishing these criteria.^14^

### 2.4 Blood cell sample collection

Peripheral venous blood samples of the patients were collected in Ethylene Diamine Tetraacetic Acid (EDTA) anticoagulant tubes after admission, and processed within 30 minutes. Blood cells were prepared by centrifugation at 3000g for 10 minutes, transferred into new tubes, and stored at −80°C until use.

### 2.5 DNA extraction

Genomic DNA was extracted from blood cell samples using DNeasy Blood Kit (Tianyihuyuan, Beijing, China), following the manufacturer's protocol.

### 2.6 Mutation sequencing

The whole exon region of LDLR, PCSK9 and LDLRAP1 genes and the region at exon 26 of APOB gene (from 100bp to 200bp of p.Arg3500), which were located at the LDLR‐binding site,^5^ were sequenced by Sanger sequencing, with the ABI 3730‐XL Genetic Analyzer employed (ABI, Foster City, California).

### 2.7 Sequence analysis and bioinformatic prediction of mutations

The LOVD database (http://www.LOVD.nl/LDLR), NCBI‐ClinVar database (https://www.ncbi.nlm.nih.gov/pmc/), and the NCBI‐Pubmed literature database (https://www.ncbi.nlm.nih.gov/pmc/) were used to determine whether the mutations were “pathogenic” or “potentially pathogenic.” If the mutation was not found in these databases, the Polyphen‐2 software (Harvard, Boston, MA) was used to analyze conservation of the amino acid caused by the mutation; if the mutation was highly conserved among different species, it was defined as pathogenic mutation.

### 2.8 Statistical analysis

The SPSS19.0 software (SPSS, Chicago, IL) was used for analysis. Measurement data of normal distribution were represented as mean ± SD and examined using independent samples *t* test. Count data were examined using *X* ^2^ test. *P* < 0.05 indicated significant difference.

## 3 RESULTS

### 3.1 Clinical characteristics and pathogenic mutations of FH

A total of 225 patients who met the inclusion criteria were enrolled in the study, including 188 males (83.6%), and 37 females (16.4%), and the average age at the first onset of MI was 46.64 ± 7.21 years old. Ten pathogenic mutations of LDLR, APOB, PCSK9 and LDLRAP1 genes were found in 11 of 225 patients, all of which were heterozygous. Among these 11 patients, there were 8 with LDLR mutation alone, 1 with APOB mutation alone, 1 with LDLRAP1 mutation alone and 1 with both LDLR (c.129G>C) and LDLRAP1 mutations (LDLRAP1 c.274G>A), without PCSK9 functional mutation. Among all mutations, 6 out of 10 mutations were classified to known “pathogenic” mutations, and other 4 mutations were classified to putative “likely pathogenic” mutations, which were newly discovered (Table 1).

**Table 1:** Pathogenic mutations of familial hypercholesterolemia in premature myocardial infarction patients

Gene	Function	cDNA position	Protein position	Significance	
LDLR	Missense	c.129G>C	p.Lys43Asn	Likely pathogenic	Putative
LDLR	Missense	c.241C>T	p.Arg81Cys	Pathogenic	Known
LDLR	Missense	c.292G>A	p.Gly98Ser	Pathogenic	Known
LDLR	Missense	c.1525A>G	p.Ile509Glu	Pathogenic	Known
LDLR	Missense	c.1691A>G	p.Ala564Ser	Pathogenic	Known
LDLR	Missense	c.1691A>G	p.Ala564Ser	Pathogenic	Known
LDLR	Missense	c.1867A>T	p.Ile623Phe	Likely pathogenic	Putative
LDLR	Missense	c.2054C>T	p.Pro685Leu	Pathogenic	Known
LDLR	Missense	c.2054C>T	p.Pro685Leu	Pathogenic	Known
Apolipoprotein B	Missense	c.10579C>T	p. Arg3527Trp	Pathogenic	Known
LDLRAP1	Missense	c.65G>C	p.Trp22Ser	Likely pathogenic	Putative
LDLRAP1	Missense	c.274G>A	p.Val92Met	Likely pathogenic	Putative

Abbreviations: LDLR, low‐density lipoprotein receptor; LDLRAP1, low‐density lipoprotein receptor adaptor protein 1.

Because LDLRAP1 mutations cause ARH, 10 patients were diagnosed as FH by genetic testing, including 8 patients with LDLR mutation, 1 with APOB mutation and 1 with LDLR and LDLRAP1 mutations. The prevalence of FH diagnosed by genetic testing was 4.4%. Compared to mutation‐negative patients, mutation‐positive patients had more severe coronary lesions, and higher LDL‐C levels (Table 2).

**Table 2:** Clinical characteristics of patients with pathogenic mutations

	Total (n = 225)	Mutation positive (n = 10)	Mutation negative (n = 215)	*P*‐value
Baseline data				
Male, n (%)	188 (83.6)	9 (90.0)	179 (83.3)	0.574
Body mass index (kg/m^2^)	26.71 ± 3.51	28.54 ± 6.40	26.62 ± 3.32	0.09
Age at the first onset of myocardial infarction (yrs)	46.64 ± 7.21	44.80 ± 6.36	46.73 ± 7.25	0.374
Gensini score	54 (34,79)	70 (53110)	54 (33,76)	0.043
Family history of premature coronary heart disease	49 (21.8)	2 (20.0)	47 (21.9)	0.889
White blood cells (10*9/L)	7.81(6.40,9.68)	7.75(6.92,9.98)	7.86 (6.30,9.50)	0.794
Hemoglobin (g/L)	141.23 ± 16.20	143.50 ± 13.01	141.13 ± 16.35	0.589
Glutamic oxalacetic aminopherase (U/L)	26 (19,44)	39 (21,73)	26 (19,44)	0.227
Glutamic‐pyruvic transaminase (U/L)	27 (18,45)	22 (18,66)	28 (18,44)	0.939
Estimated glomerular filtration rate (mL/min/1.73 m^2^)	96.72 (82.49, 104.27)	84.66 (72.81, 97.23)	97.45 (84.56, 104.59)	0.048
Risk factors				
Smoking, n (%)	153 (68.0)	10 (100.0)	143 (66.5)	0.026
Hypertension, n (%)	116 (51.6)	1 (10.0)	115 (53.5)	0.000
Diabetes, n (%)	83 (36.9)	4 (40.0)	79 (36.7)	0.835
Hyperlipidemia, n (%)	75 (33.3)	5 (50.0)	70 (32.6)	0.253
Lipid profile				
Total cholesterol (mmol/L)	4.01 (3.32,5.10)	5.04 (4.03,5.91)	3.96(3.31,5.08)	0.054
Triglyceride (mmol/L)	1.72 (1.20,2.43)	2.13 (1.14,2.54)	1.71(1.20,2.42)	0.717
High‐density lipoprotein cholesterol (mmol/L)	0.92 (0.82,1.05)	0.85 (0.79,0.98)	0.93(0.82,1.05)	0.324
LDL‐C (mmol/L)	2.47 (1.96,3.31)	3.39(2.58,4.08)	2.44(1.94,3.23)	0.037
Untreated LDL‐C (mmol/L)	3.63(2.98,4.35)	5.33(3.73,7.37)	3.62(2.96,4.29)	0.005
Drug administration				
Antiplatelet, n (%)	139 (61.8)	6 (60.0)	133 (61.9)	0.906
Calcium channel blocker, n (%)	40 (17.8)	0 (0.00)	40 (18.6)	0.133
Beta‐blocker, n(%)	92 (40.9)	5 (50.0)	87 (40.5)	0.549
Angiotensin‐converting enzyme inhibitor/angiotensin receptor blocker, n (%)	78 (34.7)	1 (10.0)	77 (35.8)	0.094
Statin, n(%)	109 (48.4)	6 (60.0)	103 (47.9)	0.454

Abbreviation: LDL‐C, low‐density lipoprotein cholesterol.

### 3.2 Clinical characteristics of patients with different gene mutations

**Figure 1:**
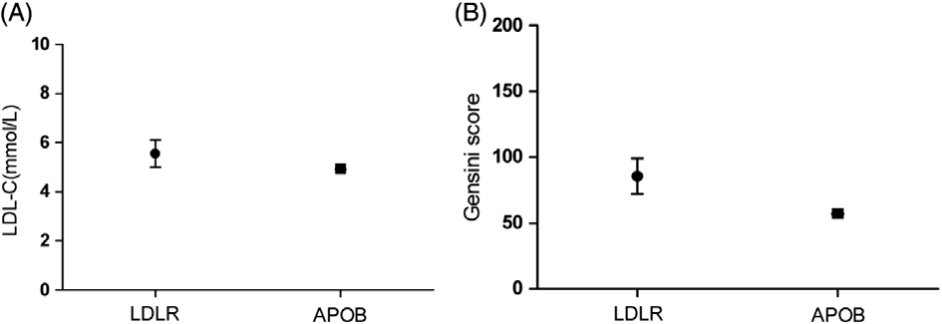
LDL‐C levels (A) and Gensini scores (B) of patients with different gene mutations. The abscissa for peer review represents different genotypes, and the ordinate represents LDL‐C levels (A) and Gensini scores (B) (n = 10). APOB, apolipoprotein B; LDL‐C, low‐density lipoprotein cholesterol; LDLR, low‐density lipoprotein receptor

Although FH cases with gene mutations generally had increased levels of LDL‐C, LDL‐C levels of individual cases with identified FH mutations were widely diverse. Only 6 out of 10 mutation‐positive patients had LDL‐C levels above 4.9 mmol/L (190 ng/dL), and LDL‐C levels were significantly higher in carriers of LDLR mutation than in those of APOB mutation (5.72 mmol/L vs 4.93 mmol/L) (Figure 1A).

Coronary angiography of 10 patients with mutation‐positive FH showed that there were three lesions in 8 patients, double‐vessel disease in 1, and a single lesion in 1. The median Gensini score of FH patients was 70, which was significantly higher than that of non‐FH patients. It was found that the median Gensini score of patients with LDLR mutation was higher than that of those with APOB mutation (78 vs 57), suggesting the patients with LDLR mutation had more severe coronary artery lesions (Figure 1B).

### 3.3 Prevalence of FH according to different diagnostic criteria

In our study, DLCN criteria and modified DLCN criteria were used for the diagnosis of FH among PMI patients, respectively, and it was found that there were 12 patients (5.3%) classified as definite or probable FH according to DLCN diagnostic criteria, and 49 patients (21.8%) diagnosed as definite or probable FH according to modified DLCN diagnostic criteria. After genetic diagnosis was introduced, the percentages of patients diagnosed as FH according to DLCN and modified DLCN criteria were 8.0% (18/225) and 23.6% (53/225), respectively.

Table 3 shows comparison of the predictive values of these two different diagnostic criteria. The diagnosis of FH based on DLCN criteria was found to have a low sensitivity, whereas that the sensitivity of diagnosis based on modified DLCN criteria was higher. On the contrary, the specificity of FH diagnosis based on DLCN criteria was high, while the diagnosis based on modified DLCN criteria had a low specificity.

**Table 3:** Sensitivity and specificity of different diagnostic criteria for familial hypercholesterolemia

Criteria	Type	Mutation rate (%)	Sensitivity (%)	Specificity (%)
DLCN	Definite + probable	4/12 (33.3)	40	96.3
Modified DLCN	Definite + probable	6/49 (12.2)	60	80

Abbreviation: DLCN, Dutch Lipid Clinic Network.

### 3.4 Treatment of FH patients

As shown in Table 4, the LDL‐C levels in PMI patients with FH (genetic testing) were far from goal attainment. Only one of FH patients had LDL‐C < 2.5 mmol/L, and none of the FH patients had LDL‐C < 1.8 mmol/L. In terms of statin use, nine patients with FH (90%) had moderate intensity medication, and only one patient with FH (10%) had high intensity medication.

**Table 4:** Treatment of FH patients

	Genetic testing for FH patients (n = 10)	Genetic testing for non‐FH patients (n = 215)	*P*‐value
Sex: male/female	9/1	179/36	0.574
Untreated LDL‐C (mmol/L)	5.33(4.24,7.37)	3.62(2.96,4.29)	0.001
Treated LDL‐C (mmol/L)	2.86(2.56,3.74)	2.12(1.80,2.60)	0.003
LDL‐C < 1.8 mmol/L, %(n)	0(0)	20.9(45)	0.106
LDL‐C < 2.5 mmol/L, %(n)	10.0(1)	64.7(139)	0.000
Cholesterol‐lowering medication			
Low intensity, %(n)	0(0)	0.5(1)	0.829
Moderate intensity, %(n)	90.0(9)	96.3(207)	0.322
High intensity, %(n)	10.0(1)	3.2(7)	0.240

Abbreviations: FH, familial hypercholesterolemia; LDL‐C, low‐density lipoprotein cholesterol.

## 4 DISCUSSION

In our study, the diagnosis of FH in patients with PMI was investigated by sequencing LDLR, APOB, PCSK9 and LDLRAP1 genes. It was found that patients with PMI showed a relatively high prevalence of mutation‐positive FH (4.4%). Compared to mutation‐negative patients, mutation‐positive patients had more severe coronary lesions and higher LDL‐C levels. LDLR mutation carriers had more severe coronary lesions than other mutation‐positive patients. Moreover, the sensitivity of modified DLCN criteria was superior to DLCN criteria.

Although the prevalence of heterozygous FH (HeFH) was estimated to be 1:500, the recent data suggested a higher prevalence, highlighting that the burden of the disease is increasing.^15^, ^16^ PMI is a great life‐threatening disease, which is considered as one of the important clinical manifestations of FH. Therefore, screening for FH among PMI patients is significant.^17^ It has been shown that the prevalence of FH with common pathogenic gene mutations in PMI patients is about 1.3% to 7.0%.^18^, ^19^ Consistent with the previous study, it was found that the prevalence of HeFH in PMI patients was 4.4%.

As one of the classic diagnostic indicators of FH, family history has been challenged. The Spanish Familial Hypercholesterolemia Cohort Study (SAFEHEART) Registry showed that family history of pCHD was present in only a minority of molecularly defined FH patients,^20^ and Séguro et al^21^ found only 18% of mutation‐positive FH patients had a personal history of pCHD. Similar results were found in our study. There was no significant difference in family history of pCHD between mutation‐positive and mutation‐negative patients, and only 20% mutation‐positive FH patients had family history of pCHD, with reduced penetrance.^22^ Their affected relatives received lipid‐lowering therapy, and the self‐reported family history may be unreliable.^23^

The DLCN criteria are often used for the diagnosis of FH. Li et al^24^ found that the prevalence of definite/probable FH in PMI patients was 7.1% based on DLCN criteria in China. However, given that lipid levels of Chinese individuals are significantly lower compared to the Western population, DLCN criteria may not be suitable for the Chinese population.^25^ Shi et al^13^ established modified DLCN criteria based on the 95th centile of LDL‐C levels in 9324 Chinese subjects, and found that the prevalence of definite/probable FH was 0.31%. Our study found that the percentage of FH based on DLCN and modified DLCN criteria among PMI patients was 8.0% and 23.6%, respectively. Moreover, modified DLCN criteria had a higher diagnostic sensitivity than DLCN criteria, indicating that modified DLCN criteria were more suitable for the Chinese population. But there are some limitations for the two diagnostic criteria, and genetic testing is still the gold standard for FH diagnosis.^8^

It has been reported that the proportion of patients with FH which could not be explained by LDLR, APOB or PCSK9 mutations was estimated to be 15.25%.^26^ Patients with clinical phenotype of FH may have negative genetic testing results for the primary genes. In our study, mutation rates of patients with definite/probable FH based on the DLCN criteria and modified DLCN criteria were only 33.3% and 12.2%, respectively. Those mutation‐negative FH patients might have a polygenic cause. Moreover, LDL‐C elevating alleles might have a cumulative effect on the FH phenotype. Since the development of the whole genome and exome sequencing, some new genes/variants have been identified, and it is considered that these newly identified genes are involved in modulating and exacerbating the phenotype of heterozygous FH.

FH patients with different genotypes showed diverse LDL‐C levels. Not all mutation‐positive patients had LDL‐C levels above a certain value. Khera et al^22^ reported that 55% of the FH patients with pathogenic variants had LDL‐C levels <190 mg/dL and 27% had LDL‐C levels <130 mg/dL. Abul‐Husn et al^27^ found that LDL‐C levels >190 mg/dL were present in 45% of the patients with FH variants. In our study, 60% of mutation‐positive patients had LDL‐C levels above 190 ng/dL. For patients with HeFH, it was found that LDL‐C levels were higher in carriers of LDLR variants compared to APOB or PCSK9 variants,^27^ consistent with our study. As shown in Figure 1, the median LDL‐C level was 5.72 mmol/L in carriers of LDLR variants, and 4.93 mmol/L in those of APOB variants. In addition, coronary lesions of LDLR variant carriers were more severe than those of APOB variant carriers.

Early treatment of FH patients is critical for improvement of prognosis. According to the guideline, the LDL‐C levels of adult FH patients should be controlled <2.5 mmol/L; if the patients were combined with CHD or diabetes, the LDL‐C level should be controlled <1.8 mmol/L.^28^ However, these FH mutation carriers are usually undertreated and have LDL‐C levels above the goal. De Luca et al^29^ used DLCN criteria to diagnose 92 definite/probable FH in 4030 patients with stable CHD, finding that a target of LDL‐C <1.8 mmol/L was reached only in 10.9% of patents with definite/probable FH. Similarly, in our study, only one FH patient had LDL‐C <2.5 mmol/L, and none of the FH patients had LDL‐C <1.8 mmol/L. Therefore, undertreatment of FH patients should arouse a widespread concern in China.

There were several limitations in our study. First, only the patients in a single center were enrolled, and the sample size was small, which may not reflect the general PMI population. Second, the new mutations we found should be verified by pedigrees or functional test. Finally, the estimated LDL‐C levels rather than the true untreated LDL‐C levels were used for the medication treated patients, which might lead to bias.

In conclusion, FH was found to be relatively common among PMI patients in China. Underdiagnosis and undertreatment of FH remain a big problem, which should arouse a widespread concern.

## CONFLICTS OF INTEREST

The authors declare no potential conflict of interests.
